# Obesity and lipid indices as predictors of depressive symptoms in middle-aged and elderly Chinese: insights from a nationwide cohort study

**DOI:** 10.1186/s12888-024-05806-z

**Published:** 2024-05-10

**Authors:** Xiaoyun Zhang, Ying Wang, Xue Yang, Yuqing Li, Jiaofeng Gui, Yujin Mei, Haiyang Liu, Lei-lei Guo, Jinlong Li, Yunxiao Lei, Xiaoping Li, Lu Sun, Liu Yang, Ting Yuan, Congzhi Wang, Dongmei Zhang, Jing Li, Mingming Liu, Ying Hua, Lin Zhang

**Affiliations:** 1 Department of Graduate School, Wannan Medical College, 22 Wenchang West Road, Higher Education Park, Wuhu City, An Hui Province People’s Republic of China; 2 Student Health Center, Wannan Medical College, 22 Wenchang West Road, Higher Education Park, Wuhu City, An Hui Province People’s Republic of China; 3 Department of Surgical Nursing, School of Nursing, Jinzhou Medical University, No.40, Section 3, Songpo Road, Linghe District, Jinzhou City, Liaoning Province People’s Republic of China; 4 Department of Occupational and Environmental Health, Key Laboratory of Occupational Health and Safety for Coal Industry in Hebei Province, School of Public Health, North China University of Science and Technology, Tangshan, Hebei Province People’s Republic of China; 5 Obstetrics and Gynecology Nursing, School of Nursing, Wannan Medical College, 22 Wenchang West Road, Higher Education Park, Wuhu City, An Hui Province People’s Republic of China; 6 Department of Emergency and Critical Care Nursing, School of Nursing, Wannan Medical College, 22 Wenchang West Road, Higher Education Park, Wuhu City, An Hui Province People’s Republic of China; 7 Department of Internal Medicine Nursing, School of Nursing, Wannan Medical College, 22 Wenchang West Road, Higher Education Park, Wuhu City, An Hui Province People’s Republic of China; 8 Department of Pediatric Nursing, School of Nursing, Wannan Medical College, 22 Wenchang West Road, Higher Education Park, Wuhu City, An Hui Province People’s Republic of China; 9 Department of Surgical Nursing, School of Nursing, Wannan Medical College, 22 Wenchang West Road, Higher Education Park, Wuhu City, An Hui Province People’s Republic of China; 10 Rehabilitation Nursing, School of Nursing, Wannan Medical College, 22 Wenchang West Road, Higher Education Park, Wuhu City, An Hui Province People’s Republic of China

**Keywords:** Depressive symptoms, Obesity, Lipid-related index, Anthropometric indicators, Middle-aged and elderly, Cohort study

## Abstract

**Background:**

Depressive symptoms are one of the most common psychiatric disorders, with a high lifetime prevalence rate among middle-aged and elderly Chinese. Obesity may be one of the risk factors for depressive symptoms, but there is currently no consensus on this view. Therefore, we investigate the relationship and predictive ability of 13 obesity- and lipid-related indices with depressive symptoms among middle-aged and elderly Chinese.

**Methods:**

The data were obtained from The China Health and Retirement Longitudinal Study (CHARLS). Our analysis includes individuals who did not have depressive symptoms at the baseline of the CHARLS Wave 2011 study and were successfully follow-up in 2013 and 2015. Finally, 3790 participants were included in the short-term (from 2011 to 2013), and 3660 participants were included in the long-term (from 2011 to 2015). The average age of participants in short-term and long-term was 58.47 years and 57.88 years. The anthropometric indicators used in this analysis included non-invasive [e.g. waist circumference (WC), body mass index (BMI), and a body mass index (ABSI)], and invasive anthropometric indicators [e.g. lipid accumulation product (LAP), triglyceride glucose index (TyG index), and its-related indices (e.g. TyG-BMI, and TyG-WC)]. Receiver operating characteristic (ROC) analysis was used to examine the predictive ability of various indicators for depressive symptoms. The association of depressive symptoms with various indicators was calculated using binary logistic regression.

**Results:**

The overall incidence of depressive symptoms was 20.79% in the short-term and 27.43% in the long-term. In males, WC [AUC = 0.452], LAP [AUC = 0.450], and TyG-WC [AUC = 0.451] were weak predictors of depressive symptoms during the short-term (*P* < 0.05). In females, BMI [AUC = 0.468], LAP [AUC = 0.468], and TyG index [AUC = 0.466] were weak predictors of depressive symptoms during the long-term (*P* < 0.05). However, ABSI cannot predict depressive symptoms in males and females during both periods (*P* > 0.05).

**Conclusion:**

The research indicates that in the middle-aged and elderly Chinese, most obesity- and lipid-related indices have statistical significance in predicting depressive symptoms, but the accuracy of these indicators in prediction is relatively low and may not be practical predictors.

## Introduction

Depressive symptoms, as one of the most common psychiatric disorders among middle-aged and elderly in worldwide, have prevalence rates of 22.1% in the USA, 34.8% in Japan, 34.6% in France, and 42.0% in China [1]. The Chinese population is aging, and it is expected that by 2050, the number of Chinese citizens aged 65 and above will reach 400 million [2]. The increased risk of depressive symptoms caused by aging is a serious problem for China [3]. A meta-analysis consisting of 32 cross-sectional studies showed that the pooled prevalence of depression symptoms among elderly people in China was 22.7%, with a higher prevalence rate among females (24.2%) than males (19.4%) and a higher prevalence rate in rural areas (29.2%) than in urban areas (20.5%) [4]. It is reported that depressive symptoms are one of the top ten causes of disability and a risk factor for a series of chronic diseases such as cardiovascular disease, diabetes, and obesity [5]. According to a population-based cohort study [6], participants with two or more depressive symptoms had 31% higher odds of having general obesity and 26% higher odds of having central obesity. Furthermore, depressive symptoms have been shown associated with a higher risk of ischemic heart disease and its subtypes [7]. It harms personal physical function and quality of life, which in turn increases the pressure on medical resources and socio-economic conditions [8].

Indeed, obesity is a common disease that may occur simultaneously with depressive symptoms [9]. According to statistics, the prevalence of overweight and obesity among Chinese adults may reach 65.3%, and the population may reach 78.995 million by 2030 [10]. As an important public health issue, research shows that obesity will increase the death probability of many diseases and lead to a series of chronic diseases (including cancer, type 2 diabetes, and dyslipidemia), which greatly affects public health and increases social and economic burden [11–14]. Body mass index (BMI) and waist circumference (WC) are the most commonly used indicators for measuring obesity. They have been used in many studies [15–17] to explore the association between obesity and some diseases (such as diabetes, metabolic syndrome, and depressive symptoms). However, BMI is only a surrogate measure of body fatness and does not distinguish body composition (muscle and fat accumulation) [18]. While waist circumference (WC) effectively reflects body size, fat percentage, and distribution, its strong correlation with BMI complicates the differentiation of their respective contributions as separate epidemiological risk factors [19, 20]. Therefore, many new obesity- and lipid-related indicators, including waist-height ratio (WHtR), visceral adiposity index (VAI), a body shape index (ABSI), body roundness index (BRI), lipid accumulation product (LAP), conicity index (CI), Chinese visceral adiposity index (CVAI), and triglyceride glucose (TyG) index have been proposed to use in epidemiological research [21–23].

Most previous studies [17, 24–32] explored the relationship between depressive symptoms and obesity, and some of them have found positive associations [17, 24, 25, 31], but others have suggested negative associations [26–29, 32], or no associations [30]. The reasons for this inconsistency may be differences in population characteristics (including age, race, and cultural differences) [33, 34], confounding factors [35], and different indices and standards for measuring obesity [36, 37]. For example, a cross-sectional study conducted based on the Mexican population aged 20 or above found that obesity measured by BMI was positively associated with depressive symptoms in Mexican women [31]. In contrast, a study report on 2604 Chinese people aged 55 and above found a negative correlation between obesity and depressive symptoms measured by BMI, supporting the “fat and jolly” hypothesis [32]. The hypothesis proposes that obesity is negatively correlated with depressive symptoms and leads to a reduction in depressive symptoms [27]. So far, these studies are not representative in predicting depressive symptoms among middle-aged and elderly people in China, as most of them only describe one indicator and do not compare it with other indicators.

It is essential to emphasize the value of surrogate obesity-related indicators as efficient, cost-effective tools for the rapid screening and preliminary identification of individuals at high risk for depressive symptoms within large populations [38–40]. Previous studies [16, 41, 42] have compared the predictive power of simple surrogate obesity-related indices (including BMI, WHtR, VAI, BRI, ABSI, LAP, and TyG index) for metabolic syndrome, and have found that LAP and TyG index have stronger predictive power than other indicators. However, few studies have comprehensively examined the association between obesity- and lipid-related indices with depressive symptoms in the Chinese population, as well as the predictive strength for depressive symptoms. Thus, the association between obesity (measured by different indices) and depressive symptoms in middle-aged and elderly Chinese has to be further researched.

The purpose of this study is to investigate the relationship between 13 obesity- and lipid-related indices and depressive symptoms based on 2-year and 4-year longitudinal data from a nationally representative sample of community-dwelling Chinese participants aged 45 years or elderly. In addition, we also compared the screening and predictive abilities of different indicators in short-term (after 2 years follow-up) and long-term follow-up periods (after 4 years follow-up), and analyzed them separately based on sex.

## Materials and methods

### Study design and participants

The China Health and Retirement Longitudinal Study (CHARLS) is a nationally representative cohort study that began in 2011 (Waves 1), targeting middle-aged and elderly people aged 45 and above in China and their spouses [43]. The participants are followed every two years through a face-to-face computer-assisted personal interview (CAPI), and data collection was carried out in 2013 (Waves 2) and 2015 (Waves 3). Data from CHARLS Waves 1, 2, and 3 were used in our study. We excluded individuals who met any of the following criteria at baseline: (1) missing data on depressive symptoms (excluding 7124 individuals) or Chinese version of the Center for Epidemiologic Studies Depression Scale (CES-D) ≥ 10 scores (excluding 7276 individuals), (2) missing data on any of the 13 indicators (excluding 3392 individuals), (3) missing data on age/sex/education levels/marital status/current residence/current smoking/alcohol consumption/taking activities/having regular exercise/chronic disease (excluding 1 individual). In addition, we excluded participants who did not have follow-up data (807 people lost to follow-up in 2013 and 937 people lost to follow-up in 2015). Finally, 3790 individuals who completed baseline surveys and short-term (from 2011–2013) follow-up surveys, and 3660 individuals who completed baseline surveys and long-term (from 2011–2015) follow-up surveys were enrolled in our research.

### Depressive symptoms assessment

The depressive symptoms in the study were assessed using the Chinese version of the Center for Epidemiologic Studies Depression Scale (CES-D) [44]. The Chinese version of CES-D consists of 10 items that are intended to reflect the severity of the depressive symptoms over the previous week. Four-points are present for each item: the total scores varied from 0 to 30, with 0 representing rarely or never (< 1 day), 1 representing sometimes or sporadically (1–2 days), 2 representing a moderate amount of the time (3–4 days) and 3 representing frequently or always (5–7 days). Participants with a higher total score may be indicated “at risk” of depressive symptoms. CES-D ≥ 10 was a better cutoff point for indicating depressive symptoms and has been reported in previous studies [45, 46]. When the CES-D value is 10, it provides the best discrimination ability when detecting individuals with or without a risk of depressive symptoms, with acceptable sensitivity, specificity, and accuracy [46]. The Chinese Version of CES-D has been confirmed to have better reliability and validity and was used frequently in predicting depressive symptoms [47].

### Anthropometric measurements

The anthropometric measurements used in this analysis included non-invasive anthropometric indicators (including WC, BMI, WHtR, ABSI, BRI, and CI) and invasive anthropometric indicators (including VAI, LAP, CVAI, TyG index, TyG-BMI, TyG-WC, and TyG-WHtR) [48–51]. These indicators are widely used as markers for obesity and insulin resistance in epidemiological studies to predict the risk of diseases (such as metabolic syndrome, depression, and diabetes) [28, 52–54]. However, most of these studies [28, 52–54] use a single indicator to study the relationship between obesity and depression, without attempting to compare the predictive power of these indicators for depression. Therefore, based on previous literature [16, 21, 24], we selected 13 obesity and lipid-related indicators to investigate their correlation with depressive symptoms. WC was measured between the iliac crest and the lower ribs on both sides, at the end of expiratory breath. BMI was measured with weight (kg) /height^2^ (m^2^) [55]. Other anthropometric measurements are calculated using the following formula. It should be noted that invasive anthropometric indicators require blood sampling to evaluate TG and HDL-C levels.

1. $${\text{WHtR}}=\mathrm{WC }\left({\text{cm}}\right) /\mathrm{ Height} \left({\text{cm}}\right)$$ [56]
2. Males: $${\text{VAI}}=\frac{WC\left(cm\right)}{39.68+\left(1.88\times BMI\right)}\times \frac{TG\left(mmol/l\right)}{1.03}\times \frac{1.31}{HDL-C\left(mmol/l\right)}$$ [51]
  Females: $${\text{VAI}}=\frac{WC\left(cm\right)}{36.58+\left(1.89\times BMI\right)}\times \frac{TG\left(mmol/l\right)}{0.81}\times \frac{1.52}{HDL-C\left(mmol/l\right)}$$
3. $${\text{ABSI}}=\frac{WC(m)}{{{Height(m)}^{1/2}\times BMI}^{2/3}}$$ [56]
4. $${\text{BRI}}=364.2-365.5\sqrt{1-(\frac{(WC(m)/{(2\uppi ))}^{2}}{{\left(0.5\times Height(m)\right)}^{2}})}$$ [57]
5. Males: $${\text{LAP}}=\left[\mathrm{WC }\left({\text{cm}}\right)-65\right]\times \mathrm{TG }\left({\text{mmo}}1/1\right)$$ [21]
  Females: $${\text{LAP}}=\left[\mathrm{WC }\left({\text{cm}}\right)-58\right]\times \mathrm{TG }\left({\text{mmo}}1/1\right)$$
6. $${\text{CI}}=\frac{WC\left(m\right)}{0.109\sqrt{\frac{weight\left(kg\right)}{height(m)}}}$$ [23]
7. Males: $${\text{CVAI}}=-267.93+0.68\times {\text{age}}+0.03\times \mathrm{BMI }\left({\text{kg}}/{{\text{m}}}^{2}\right) +4.00\times \mathrm{WC }\left({\text{cm}}\right)+22.00\times {{\text{log}}}_{10}{\text{TG}} \left({\text{mmo}}1/1\right)-16.32\times {\text{HDL}}-{\text{C}} \left({\text{mmo}}1/1\right)$$ [51]
  Females: $${\text{CVAI}}=-187.32+1.71\times {\text{age}}+4.32\times \mathrm{BMI }\left({\text{kg}}/{{\text{m}}}^{2}\right) +1.12\times \mathrm{WC }\left({\text{cm}}\right)+39.76\times {{\text{log}}}_{10}{\text{TG}} \left({\text{mmo}}1/1\right)-11.66\times {\text{HDL}}-{\text{C}} \left({\text{mmo}}1/1\right)$$
8. $$\mathrm{TyG index}={\text{Ln}}\left[\left({\text{TG}}\left({\text{mg}}/{\text{dl}}\right)\times \mathrm{glucose }\left({\text{mg}}/{\text{dl}}\right)/2\right)\right]$$ [21]
9. $${\text{TyG}}-{\text{BMI}}={\text{TyG}}\times {\text{BMI}}$$ [50]
10. $${\text{TyG}}-{\text{WC}}={\text{TyG}}\times {\text{WC}}$$ [50]
11. $${\text{TyG}}-{\text{WHtR}}={\text{TyG}}\times {\text{WHtR}}$$ [50]

### Covariates

Socio-demographic characteristics include age, sex (1 = male, 2 = female), education levels, marital status, current residence, current smoking, alcohol consumption, taking activities, having regular exercise, and chronic disease. (1) age was sorted as four categories: 45–54, 55–64, 65–74, and above 75 years old; (2) education levels were classified into four groups: illiterate, less than elementary school, high school, and above vocational school; (3) marital status was classified into two categories: single and married; (4) current residence included the urban and rural; (5) current smoking was categorized into three groups: no smoker, former smoker and current smoker; (6) alcohol consumption was divided into three groups, which included never drinking, less than once a month, and more than once a month; (7) taking activities were sorted as two groups: the ever (at least once a month) and never; (8) having regular exercise included no exercise, less than exercises, and regular exercises; (9) the counts of chronic disease were classified into 0, 1–2, 3–14. Chronic diseases in our study, including hypertension, dyslipidemia, diabetes or hyperglycemia, malignant tumor, chronic lung disease, liver disease, heart disease, stroke, kidney disease, stomach or digestive system disease, mental and emotional diseases, memory-related diseases, arthritis or rheumatism, asthma. The presence of each disease is rated as 1, and the total score for all diseases ranges from 0 to 14. In terms of the number of chronic diseases, participants with three or more chronic diseases have a higher risk of depressive symptoms compared to those without any chronic disease [58]. These categories have been used extensively in our previous research [59–63].

### Statistical analysis

Mean and standard deviation were used to express continuous variables. Categorical variables were expressed as frequencies and percentages. In order to calculate the differences in mean distribution by sex and with or without depressive symptoms, independent sample t-tests were utilized. Socio-demographic characteristics were categorized by sex and presented as frequencies and percentages. Differences between the male and female groups were tested for statistical significance using the Chi-square test. Binary logistic regression analysis was used to evaluate the associations between obesity- and lipid-related indices and depressive symptoms, with 13 indices as independent variables and depressive symptoms as dependent variables. Adjusting for age, sex, education levels, marital status, current residence, current smoking, alcohol consumption, taking activities, having regular exercise, and chronic disease, we reported odds ratios (ORs) and 95% confidence intervals (CIs). The receiver operating characteristic curve (ROC) was utilized to evaluate the performance of obesity- and lipid-related indices as predictors of depressive symptoms. The area under curve (AUC) and its 95% CIs were calculated to quantify this performance. The significance of the AUC is that an area greater than 0.9 indicates high accuracy, 0.71–0.9 indicates moderate accuracy and 0.5–0.7 indicates low accuracy [64]. Our data satisfies three assumptions required for statistical testing: normality, homogeneity of variance, and data independence. All of the statistical analyses were analyzed using the IBM SPSS version 25.0 (IBM Corp., Armonk, NY). *P* < 0.05 was considered statistically significant in all the analyses.

## Results

Table 1 showed the basic characteristics of the study participants. A total of 3790 participants were included in the short-term (2 years from 2011 to 2013) and 3660 in the long-term (4 years from 2011 to 2015). For the missing data, we found that there was no difference in socio-demographic characteristics compared to all the data, so we adopted a direct deletion method for the missing data. At baseline, 53.54% of the participants were males in the short-term, and 53.63% males in the long-term. The mean BMI, WHtR, VAI, ABSI, BRI, LAP, CI, CVAI, TyG index, TyG-BMI, TyG-WC and TyG-WHtR in females are higher than males during short- and long-term (*P* < 0.05). During both short-term and long-term, we also observed the significant differences in age, education levels, marital status, current smoking, alcohol consumption between males and females, but observed no significant differences in the distribution of current residence, taking activities, and having regular exercises.

**Table 1:** Baseline characteristics of participants with full samples

Characteristics	2011 → 2013 (*N* = 3790)				2011 → 2015 (*N* = 3660)			
	**Male**	**Female**	***t/χ***^***2***^	***P***	**Male**	**Female**	***t/χ***^***2***^	***P***
	**N (%)**	**N (%)**			**N (%)**	**N (%)**		
N	2029(53.54)	1761(46.46)			1963(53.63)	1697(46.37)		
Age(years)								
45–54	616(30.36)	733(41.62)	56.811	0.000	635(32.35)	738(43.49)	51.188	0.000
55–64	851(41.94)	657(37.31)			840(42.79)	629(37.07)		
65–74	432(21.29)	297(16.87)			387(19.71)	274(16.15)		
≥ 75	130(6.41)	74(4.20)			101(5.15)	56(3.30)		
Education								
Illiterate	232(11.43)	622(35.32)	318.401	0.000	206(10.49)	582(34.30)	323.351	0.000
Less than elementary school	1466(72.25)	973(55.25)			1434(73.05)	970(57.16)		
High school	211(10.40)	120(6.81)			209(10.65)	109(6.42)		
Above vocational school	120(5.91)	46(2.61)			114(5.81)	36(2.12)		
Marital status								
Single	122(6.01)	194(11.02)	30.884	0.000	121(6.16)	175(10.31)	21.070	0.000
Married	1907(93.99)	1567(88.98)			1842(93.84)	1522(89.69)		
Current residence								
Rural	1848(91.08)	1589(90.23)	0.800	0.371	1798(91.59)	1539(90.69)	0.927	0.336
Urban	181(8.92)	172(9.77)			165(8.41)	158(9.31)		
Current smoking								
No	523(25.78)	1657(94.09)	1800.817	0.000	512(26.08)	1600(94.28)	1734.818	0.000
Former smoker	340(16.76)	23(1.31)			310(15.79)	17(1.00)		
Current smoker	1166(57.47)	81(4.60)			1141(58.13)	80(4.71)		
Alcohol consumption								
No	855(42.14)	1562(88.70)	910.655	0.000	805(41.01)	1514(89.22)	929.674	0.000
Less than once a month	220(10.84)	83(4.71)			223(11.36)	73(4.30)		
More than once a month	954(47.02)	116(6.59)			935(47.63)	110(6.48)		
Taking activities								
No	940(46.33)	813(46.17)	0.010	0.921	885(45.08)	792(46.67)	0.923	0.337
Yes	1089(53.67)	948(53.83)			1078(54.92)	905(53.33)		
Having regular exercises								
No exercise	1224(60.33)	1061(60.25)	3.440	0.179	1187(60.47)	1022(60.22)	2.664	0.264
Less than exercises	410(20.21)	323(18.34)			393(20.02)	313(18.44)		
Regular exercises	395(19.47)	377(21.41)			383(19.51)	362(21.33)		
Chronic diseases(counts)								
0	808(39.82)	639(36.29)	6.226	0.044	784(39.94)	636(37.48)	2.977	0.226
1–2	975(48.05)	875(49.69)			942(47.99)	833(49.09)		
3–14	246(12.12)	247(14.03)			237(12.07)	228(13.44)		
WC (cm)	85.95 ± 9.66	86.04 ± 9.86	-0.286	0.775	85.95 ± 9.68	86.21 ± 9.96	-0.819	0.413
BMI (kg/m^2^)	23.34 ± 3.60	24.35 ± 4.12	-7.998	0.000	23.40 ± 3.59	24.39 ± 4.00	-7.889	0.000
WHtR	0.52 ± 0.06	0.56 ± 0.07	-18.960	0.000	0.52 ± 0.06	0.56 ± 0.07	-18.973	0.000
VAI	4.05 ± 4.25	6.32 ± 6.07	-13.095	0.000	4.05 ± 4.30	6.27 ± 6.15	-12.457	0.000
ABSI	8.24 ± 0.50	8.31 ± 0.61	-4.061	0.000	8.22 ± 0.51	8.31 ± 0.60	-4.746	0.000
BRI	3.86 ± 1.14	4.67 ± 1.47	-18.637	0.000	3.86 ± 1.14	4.68 ± 1.48	-18.658	0.000
LAP	32.85 ± 33.76	45.70 ± 37.17	-11.068	0.000	32.87 ± 33.67	45.55 ± 37.22	-10.737	0.000
CI	1.28 ± 0.08	1.30 ± 0.09	-7.049	0.000	1.27 ± 0.08	1.30 ± 0.09	-7.686	0.000
CVAI	100.07 ± 47.24	107.85 ± 44.04	-5.245	0.000	99.56 ± 47.52	107.02 ± 43.83	-4.941	0.000
TyG index	8.64 ± 0.65	8.75 ± 0.65	-5.258	0.000	8.64 ± 0.65	8.73 ± 0.64	-4.422	0.000
TyG-BMI	202.38 ± 39.32	213.70 ± 42.76	-8.434	0.000	202.84 ± 39.37	213.59 ± 41.47	-8.034	0.000
TyG-WC	744.84 ± 117.72	754.84 ± 116.51	-2.621	0.009	744.68 ± 117.96	754.75 ± 116.83	-2.585	0.010
TyG -WHtR	4.53 ± 0.69	4.92 ± 0.77	-16.397	0.000	4.53 ± 0.69	4.92 ± 0.77	-16.020	0.000

*WC* waist circumference, *BMI* body mass index, *WHtR* waist to height ratio, *VAI* visceral adiposity index, *ABSI* A body shape index, *BRI* body roundness index, *LAP* lipid accumulation product, *CVAI* Chinese visceral adiposity index, *CI* conicity index, *TyG index* triglyceride glucose index, *TyG-BMI* TyG related to BMI, *TyG-WC* TyG related to WC, *TyG-WHtR* TyG related to WHtR

Table 2 showed the baseline characteristics of the study participants with and without depressive symptoms by sex at 2011 → 2013. After 2 years follow-up, approximately 20.79% of the participants had depressive symptoms (16.76% in males and 25.44% in females). Males with depressive symptoms had significant differences in current residence, current smoking, WC, WHtR, VAI, BRI, LAP, CI, CVAI, TyG-BMI, TyG-WC, and TyG-WHtR (*P* < 0.05) during the short-term follow-up. Females with depressive symptoms had significant differences in current residence and chronic diseases (*P* < 0.05).

**Table 2:** Characteristics of the study participants with and without depressive symptoms by sex at 2011 → 2013

Variables	Male (*N* = 2029)		*χ2*	*P*	Female (*N* = 1761)		*χ2*	*P*
	**Depressive symptoms N (%)**	**No-depressive symptoms N (%)**			**Depressive symptoms N (%)**	**No-depressive symptoms N (%)**		
N	340(16.76)	1689(83.24)			448(25.44)	1313(74.56)		
Age(years)								
45–54	106(31.18)	510(30.20)	2.272	0.518	183(40.85)	550(41.89)	1.819	0.611
55–64	133(39.12)	718(42.51)			178(39.73)	479(36.48)		
65–74	81(23.82)	351(20.78)			70(15.63)	227(17.29)		
≥ 75	20(5.88)	110(6.51)			17(3.79)	57(4.34)		
Education								
Illiterate	48(14.12)	184(10.89)	6.008	0.111	163(36.38)	459(34.96)	3.339	0.342
Less than elementary school	249(73.24)	1217(72.05)			251(56.03)	722(54.99)		
High school	27(7.94)	184(10.89)			27(6.03)	93(7.08)		
Above vocational school	16(4.71)	104(6.16)			7(1.56)	39(2.97)		
Marital status								
Single	23(6.76)	99(5.86)	0.409	0.523	60(13.39)	134(10.21)	3.461	0.063
Married	317(93.24)	1590(94.14)			388(86.61)	1179(89.79)		
Current residence								
Rural	325(95.59)	1523(90.17)	10.220	0.001	424(94.64)	1165(88.73)	13.259	0.000
Urban	15(4.41)	166(9.83)			24(5.36)	148(11.27)		
Current smoking								
No	90(26.47)	433(25.64)	8.387	0.015	416(92.86)	1241(94.52)	4.170	0.124
Former smoke	39(11.47)	301(17.82)			10(2.23)	13(0.99)		
Current smoke	211(62.06)	955(56.54)			22(4.91)	59(4.49)		
Alcohol consumption								
No	140(41.18)	715(42.33)	1.861	0.394	404(90.18)	1158(88.19)	1.357	0.507
Less than once a month	44(12.94)	176(10.42)			19(4.24)	64(4.87)		
More than once a month	156(45.88)	798(47.25)			25(5.58)	91(6.93)		
Taking activities								
No	173(50.88)	767(45.41)	3.407	0.065	216(48.21)	597(45.47)	1.013	0.314
Yes	167(49.12)	922(54.59)			232(51.79)	716(54.53)		
Having regular exercises								
No exercise	210(61.76)	1014(60.04)	1.171	0.557	258(57.59)	803(61.16)	1.940	0.379
Less than exercises	71(20.88)	339(20.07)			90(20.09)	233(17.75)		
Regular exercises	59(17.35)	336(19.89)			100(22.32)	277(21.10)		
Chronic diseases(counts)								
0	138(40.59)	670(39.67)	0.205	0.903	122(27.23)	517(39.38)	24.171	0.000
1–2	163(47.94)	812(48.08)			244(54.46)	631(48.06)		
3–14	39(11.47)	207(12.26)			82(18.30)	165(12.57)		
WC (cm)	84.62 ± 9.33	86.22 ± 9.70	2.787	0.005	85.36 ± 9.67	86.27 ± 9.92	1.685	0.092
BMI (kg/m^2^)	23.01 ± 3.47	23.40 ± 3.62	1.830	0.067	24.23 ± 4.99	24.39 ± 3.77	0.615	0.539
WHtR	0.52 ± 0.05	0.52 ± 0.06	2.313	0.021	0.56 ± 0.07	0.56 ± 0.07	0.677	0.498
VAI	3.63 ± 4.13	4.14 ± 4.27	2.052	0.041	6.23 ± 6.15	6.35 ± 6.05	0.355	0.732
ABSI	8.20 ± 0.55	8.24 ± 0.49	1.392	0.164	8.32 ± 0.67	8.31 ± 0.59	-0.397	0.691
BRI	3.73 ± 1.09	3.89 ± 1.15	2.303	0.021	4.63 ± 1.56	4.68 ± 1.44	0.591	0.555
LAP	28.71 ± 30.96	33.69 ± 34.25	2.656	0.008	44.07 ± 34.79	46.25 ± 37.94	1.074	0.283
CI	1.27 ± 0.08	1.28 ± 0.08	2.149	0.032	1.29 ± 0.09	1.30 ± 0.09	0.278	0.781
CVAI	93.33 ± 45.03	101.43 ± 47.57	2.998	0.003	106.08 ± 43.49	108.46 ± 44.22	0.984	0.325
TyG index	8.59 ± 0.60	8.65 ± 0.66	1.760	0.079	8.75 ± 0.65	8.75 ± 0.64	0.204	0.838
TyG-BMI	198.19 ± 36.79	203.23 ± 39.77	2.157	0.031	212.53 ± 49.18	214.09 ± 40.35	0.606	0.545
TyG-WC	728.60 ± 111.26	748.11 ± 118.74	2.917	0.004	748.22 ± 113.21	757.10 ± 117.57	1.394	0.164
TyG -WHtR	4.45 ± 0.66	4.55 ± 0.70	2.566	0.011	4.90 ± 0.76	4.93 ± 0.77	0.679	0.497

*WC* waist circumference, *BMI* body mass index, *WHtR* waist to height ratio, *VAI* visceral adiposity index, *ABSI* A body shape index, *BRI* body roundness index, *LAP* lipid accumulation product, *CVAI* Chinese visceral adiposity index, *CI* conicity index, *TyG* triglyceride glucose index, *TyG-BMI* TyG related to BMI, *TyG-WC* TyG related to WC, *TyG-WHtR* TyG related to WHtR

Table 3 showed the baseline characteristics of the study participants with and without depressive symptoms by sex at 2011 → 2015. After 4 years follow-up, approximately 27.43% of the participants had depressive symptoms (21.50% in males and 34.30% in females). Marital status was significantly different between males with and without depressive symptoms during the long-term follow-up (*P* < 0.05). Females with depressive symptoms had significant differences in current residence, taking activities, chronic diseases, WC, BMI, BRI, CVAI, TyG index, TyG-BMI, TyG-WC, and TyG-WHtR (*P* < 0.05).

**Table 3:** Characteristics of the study participants with and without depressive symptoms by sex at 2011 → 2015

Variables	Male (*N* = 1963)		*χ2*	*P*	Female (*N* = 1697)		*χ2*	*P*
**N (%)**	**Depressive symptoms N (%)**	**No-depressive symptoms N (%)**			**Depressive symptoms N (%)**	**No-depressive** **Symptoms N (%)**		
N	422(21.50)	1541(78.50)			582(34.30)	1115(65.70)		
Age (years)								
45–54	137(32.46)	498(32.32)	1.424	0.700	262(45.02)	476(42.69)	1.902	0.593
55–64	188(44.55)	652(42.31)			217(37.29)	412(36.95)		
65–74	75(17.77)	312(20.25)			86(14.78)	188(16.86)		
≥ 75	22(5.21)	79(5.13)			17(2.92)	39(3.50)		
Education								
Illiterate	56(13.27)	150(9.73)	6.220	0.101	202(34.71)	380(34.08)	0.303	0.960
Less than elementary school	307(72.75)	1127(73.13)			328(56.36)	642(57.58)		
High school	40(9.48)	169(10.97)			39(6.70)	70(6.28)		
Above vocational school	19(4.50)	95(6.16)			13(2.23)	23(2.06)		
Marital status								
Single	35(8.29)	86(5.58)	4.216	0.040	61(10.48)	114(10.22)	0.027	0.869
Married	387(91.71)	1455(94.42)			521(89.52)	1001(89.78)		
Current residence								
Rural	395(93.60)	1403(91.04)	2.814	0.093	543(93.30)	996(89.33)	7.144	0.008
Urban	27(6.40)	138(8.96)			39(6.70)	119(10.67)		
Current smoking								
No	97(22.99)	415(26.93)	2.733	0.255	549(94.33)	1051(94.26)	0.472	0.790
Former smoke	71(16.82)	239(15.51)			7(1.20)	10(0.90)		
Current smoke	254(60.19)	887(57.56)			26(4.47)	54(4.84)		
Alcohol consumption								
No	175(41.47)	630(40.88)	5.987	0.050	512(87.97)	1002(89.87)	2.981	0.225
Less than once a month	61(14.45)	162(10.51)			24(4.12)	49(4.39)		
More than once a month	186(44.08)	749(48.60)			46(7.90)	64(5.74)		
Taking activities								
No	200(47.39)	685(44.45)	1.158	0.282	291(50.00)	501(44.93)	3.945	0.047
Yes	222(52.61)	856(55.55)			291(50.00)	614(55.07)		
Having regular exercises								
No exercise	261(61.85)	926(60.09)	0.435	0.805	359(61.68)	663(59.46)	1.095	0.578
Less than exercises	82(19.43)	311(20.18)			100(17.18)	213(19.10)		
Regular exercises	79(18.72)	304(19.73)			123(21.13)	239(21.43)		
Chronic diseases (counts)								
0	158(37.44)	626(40.62)	2.902	0.234	192(32.99)	444(39.82)	8.566	0.014
1–2	204(48.34)	738(47.89)			300(51.55)	533(47.80)		
3–14	60(14.22)	177(11.49)			90(15.46)	138(12.38)		
WC (cm)	85.43 ± 9.96	86.09 ± 9.60	1.236	0.261	85.32 ± 9.72	86.68 ± 10.05	2.683	0.007
BMI (kg/m^2^)	23.19 ± 3.65	23.45 ± 3.58	1.351	0.177	24.03 ± 3.53	24.58 ± 4.21	2.881	0.004
WHtR	0.52 ± 0.06	0.52 ± 0.06	0.620	0.535	0.56 ± 0.06	0.56 ± 0.07	1.963	0.050
VAI	4.24 ± 4.58	4.00 ± 4.22	-1.002	0.317	6.01 ± 6.17	6.40 ± 6.13	1.255	0.210
ABSI	8.24 ± 0.53	8.22 ± 0.50	-0.667	0.505	8.31 ± 0.57	8.31 ± 0.61	0.053	0.957
BRI	3.83 ± 1.17	3.87 ± 1.13	0.543	0.587	4.58 ± 1.36	4.73 ± 1.53	2.024	0.043
LAP	33.63 ± 35.74	32.66 ± 33.09	-0.523	0.601	43.19 ± 35.60	46.78 ± 38.00	1.885	0.060
CI	1.27 ± 0.08	1.27 ± 0.08	-0.043	0.966	1.29 ± 0.09	1.30 ± 0.09	0.954	0.340
CVAI	98.05 ± 48.76	99.97 ± 47.19	0.737	0.461	102.57 ± 42.64	109.35 ± 44.28	3.032	0.002
TyG index	8.64 ± 0.69	8.64 ± 0.64	-0.240	0.810	8.69 ± 0.64	8.75 ± 0.64	2.044	0.041
TyG-BMI	201.33 ± 41.25	203.26 ± 38.84	0.891	0.373	209.35 ± 38.15	215.81 ± 42.95	3.051	0.002
TyG-WC	741.09 ± 122.64	745.67 ± 116.67	0.705	0.481	743.34 ± 116.01	760.70 ± 116.86	2.912	0.004
TyG -WHtR	4.52 ± 0.72	4.53 ± 0.69	0.272	0.786	4.86 ± 0.76	4.95 ± 0.77	2.388	0.017

*WC* waist circumference, *BMI* body mass index, *WHtR* waist to height ratio, *VAI* visceral adiposity index, *ABSI* A body shape index, *BRI* body roundness index, *LAP* lipid accumulation product, *CVAI* Chinese visceral adiposity index, *CI* conicity index, *TyG* triglyceride glucose index, *TyG-BMI* TyG related to BMI, *TyG-WC* TyG related to WC, *TyG-WHtR* TyG related to WHtR

Table 4 showed the associations of obesity- and lipid-related indices with depressive symptoms. We use these indicators as continuous variables and depression as a binary variable, and the results are explained as how much the risk of depressive symptoms decreases or increases for every 1 unit increase in the indicators. In males, after controlling for age, educational levels, marital status, current residence, current smoking, alcohol consumption, taking activities, having regular exercises, and chronic diseases, WC (OR = 0.987, 95%CI: 0.974–1.000), LAP (OR = 0.996, 95%CI: 0.992–1.000), CVAI (OR = 0.997, 95%CI: 0.995–1.000), TyG-WC (OR = 0.999, 95%CI: 0.998–1.000) was significantly correlated with depressive symptoms during the short-term (*P* < 0.05). For example, for every unit increase in WC and TyG-WC, the risk of depressive symptoms decreases by 0.013 and 0.001 times, respectively. In females, WC (OR = 0.983, 95%CI: 0.973–0.993), BMI (OR = 0.953, 95%CI: 0.926–0.979), WHtR (OR = 0.130, 95%CI: 0.026–0.647), BRI (OR = 0.908, 95%CI: 0.843–0.978), LAP (OR = 0.997, 95%CI: 0.994–1.000), CVAI (OR = 0.996, 95%CI: 0.993–0.998), TyG index (OR = 0.834, 95%CI: 0.708–0.983), TyG-BMI (OR = 0.995, 95%CI: 0.992–0.998), TyG-WC (OR = 0.998, 95%CI: 0.998–0.999), and TyG-WHtR (OR = 0.814, 95%CI: 0.707–0.936) were significantly associated with depressive symptoms during the long-term (*P* < 0.05). For every unit increase in BMI and TyG-index, the risk of depressive symptoms decreases by 0.047 and 0.166 times, respectively. There were no significant associations between ABSI and depressive symptoms in males and females during both follow-up periods (*P* > 0.05).

**Table 4:** Associations of obesity- and lipid-related indices with depressive symptom

**Follow-up periods**		**WC**	**BMI**	**WHtR**	**VAI**	**ABSI**	**BRI**
**2011 → 2013**	**Male**						
***N* = 3790**	Unadjusted OR (95% CI)	0.983(0.970,0.995^)*^	0.969(0.936,1.002)	0.083(0.010,0.687)^*^	0.967(0.937,0.999)^*^	0.848(0.672,1.070)	0.883(0.794,0.982)^*^
	Adjusted OR (95% CI)	0.987(0.974,1.000)^*^	0.981(0.946,1.017)	0.151(0.017,1.346)	0.972(0.941,1.004)	0.835(0.656,1.062)	0.909(0.815,1.015)
	**Female**						
	Unadjusted OR (95% CI)	0.991(0.980,1.002)	0.991(0.965,1.017)	0.570(0.112,2.896)	0.997(0.979,1.015)	1.036(0.869,1.235)	0.978(0.908,1.053)
	Adjusted OR (95% CI)	0.985(0.974,0.996)^*^	0.979(0.952,1008)	0.222(0.040,1.237)	0.995(0.977,1.014)	1.003(0.824,1.220)	0.937(0.865,1.014)
**2011 → 2015**	**Male**						
***N* = 3660**	Unadjusted OR (95% CI)	0.993(0.982,1.004)	0.979(0.949,1.010)	0.546(0.080,3.702)	1.012(0.988,1.037)	1.075(0.869,1.329)	0.974(0.886,1.071)
	Adjusted OR (95% CI)	0.995(0.983,1.006)	0.981(0.949,1.014)	0.669(0.092,4.854)	1.013(0.989,1.038)	1.096(0.880,1.365)	0.983(0.891,1.085)
	**Female**						
	Unadjusted OR (95% CI)	0.986(0.976,0.996)^*^	0.964(0.939,0.990)^*^	0.218(0.048,1.001)	0.989(0.973,1.006)	0.995(0.841,1.178)	0.93(0.867,0.998)^*^
	Adjusted OR (95% CI)	0.983(0.973,0.993)^*^	0.953(0.926,0.979)^*^	0.130(0.026,0.647)^*^	0.988(0.971,1.005)	1.016(0.845,1.222)	0.908(0.843,0.978)^*^

**Follow-up periods**	**LAP**	**CI**	**CVAI**	**TyG index**	**TyG-BMI**	**TyG-WC**	**TyG -WHtR**
**2011 → 2013**							
***N* = 3790**	0.995(0.991,0.999)^*^	0.200(0.046,0.870)^*^	0.996(0.994,0.999)^*^	0.858(0.715,1.031)	0.997(0.993,1.000)^*^	0.999(0.998,1.000)^*^	0.804(0.675,0.956)^*^
	0.996(0.992,1.000)^*^	0.238(0.053,1.071)	0.997(0.995,1.000)^*^	0.888(0.737,1.071)	0.998(0.994,1.001)	0.999(0.998,1.000)^*^	0.844(0.704,1.010)
	0.998(0.995,1.001)	0.849(0.268,2.691)	0.999(0.996,1.001)	0.983(0.832,1.161)	0.999(0.997,1.002)	0.999(0.998,1.000)	0.953(0.828,1.096)
	0.997(0.994,1.001)	0.520(0.147,1.839)	0.998(0.995,1.000)	0.956(0.805,1.137)	0.998(0.995,1.001)	0.999(0.998,1.000)^*^	0.884(0.761,1.026)
**2011 → 2015**							
***N* = 3660**	1.001(0.998,1.004)	1.030(0.269,3.945)	0.999(0.997,1001)	1.021(0.866,1.204)	0.999(0.996,1.002)	1.000(0.999,1.001)	0.979(0.837,1.144)
	1.001(0.998,1.004)	1.215(0.308,4.803)	1.000(0.997,1.002)	1.029(0.870,1.219)	0.999(0.996,1.002)	1.000(0.999,1.001)	0.993(0.845,1.167)
	0.997(0.994,1.000)	0.588(0.197,1.751)	0.996(0.994,0.999)^*^	0.847(0.723,0.994)^*^	0.996(0.994,0.999)^*^	0.999(0.998,1.000)^*^	0.852(0.746,0.972)^*^
	0.997(0.994,1.000)^*^	0.534(0.164,1.736)	0.996(0.993,0.998)^*^	0.834(0.708,0.983)^*^	0.995(0.992,0.998)^**^	0.998(0.998,0.999)^*^	0.814(0.707,0.936)^*^

*WC* waist circumference, *BMI* body mass index, *WHtR* waist to height ratio, *VAI* visceral adiposity index, *ABSI* A body shape index, *BRI* body roundness index, *LAP* lipid accumulation product, *CVAI* Chinese visceral adiposity index, *CI* conicity index, *TyG* triglyceride glucose index, *TyG-BMI* TyG related to BMI, *TyG-WC* TyG related to WC, *TyG-WHtR* TyG related to WHtR

Odds ratios were adjusted for age, educational levels, marital status, current residence, current smoking, alcohol consumption, taking activities, having regular exercises, chronic diseases

^*^P < 0.05

^**^P < 0.001

**Fig. 1:**
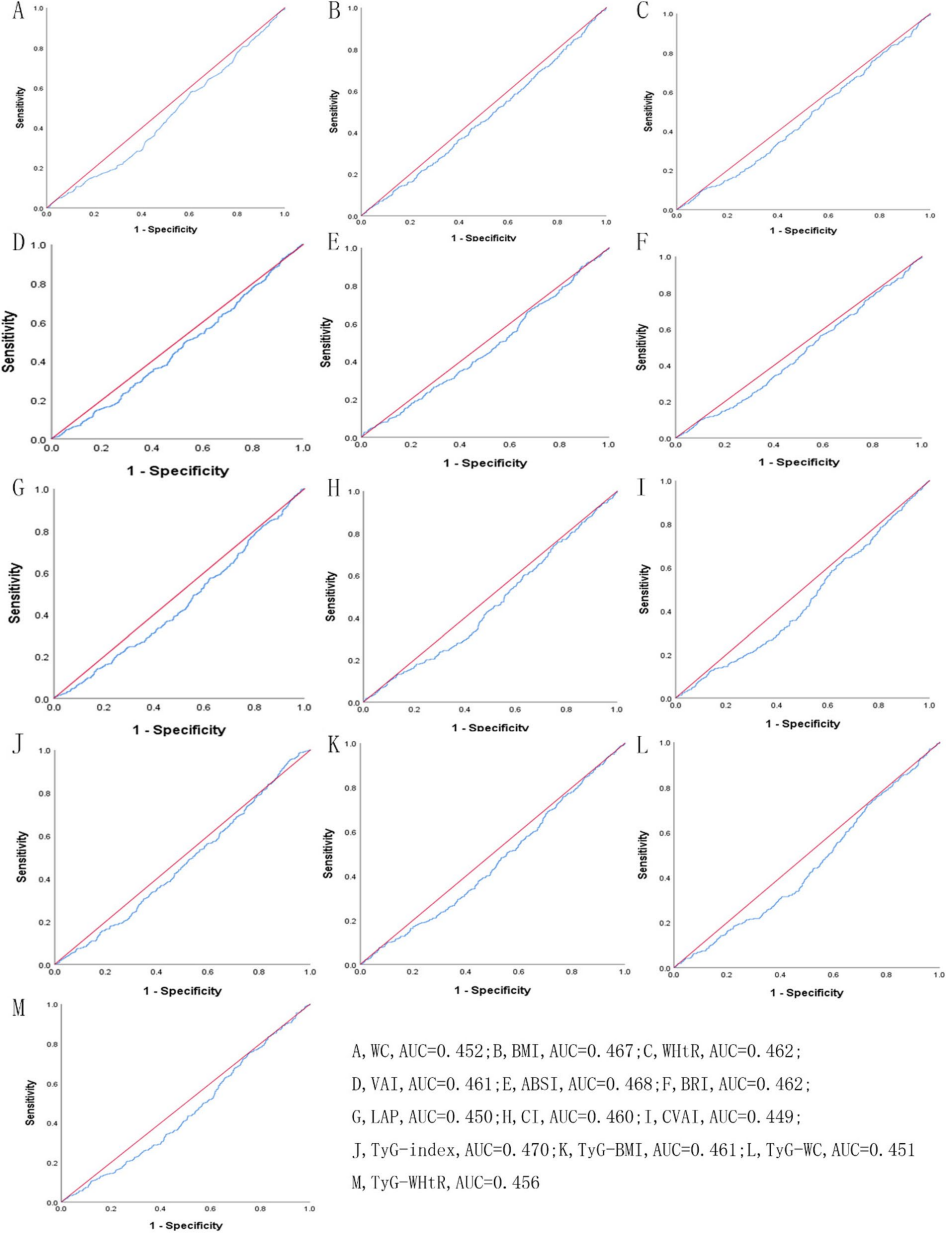
^The ROC curves of each indicator in the prediction of depressive symptoms risk in males at 2011→2013^

**Fig. 2:**
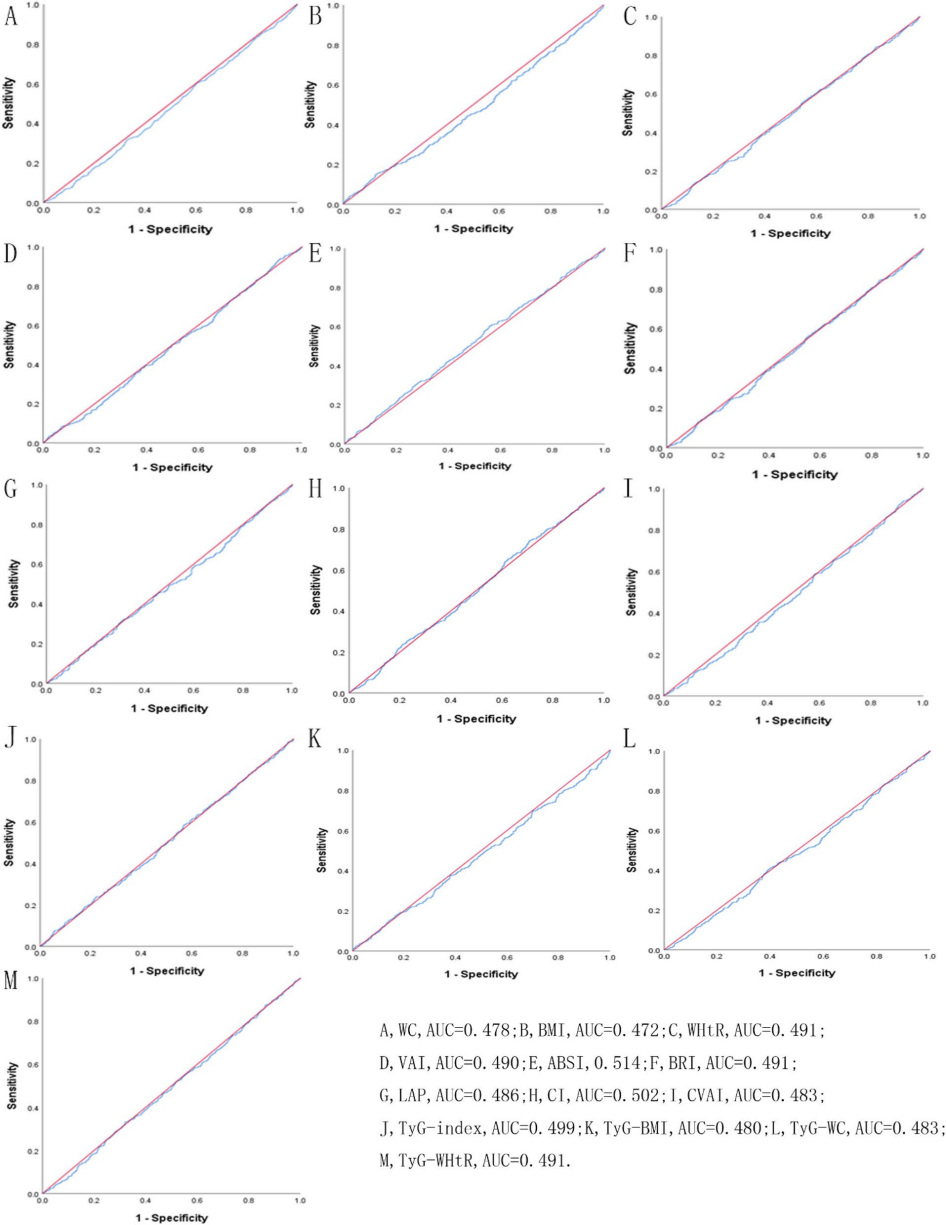
^The ROC curves of each indicator in the prediction of depressive symptoms risk in females at 2011→2013^

**Fig. 3:**
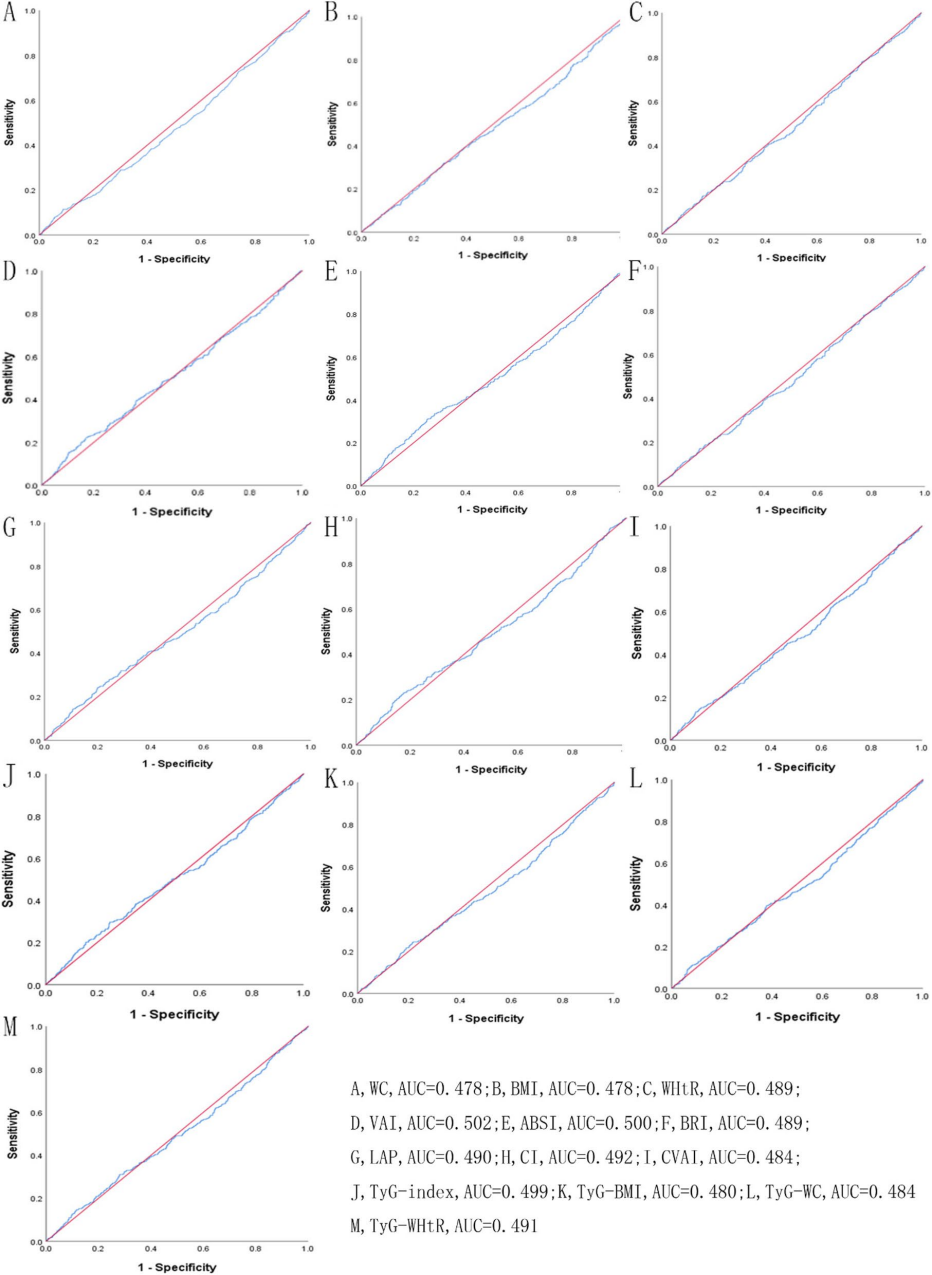
^The ROC curves of each indicator in the prediction of depressive symptoms risk in males at 2011→2015^

**Fig. 4:**
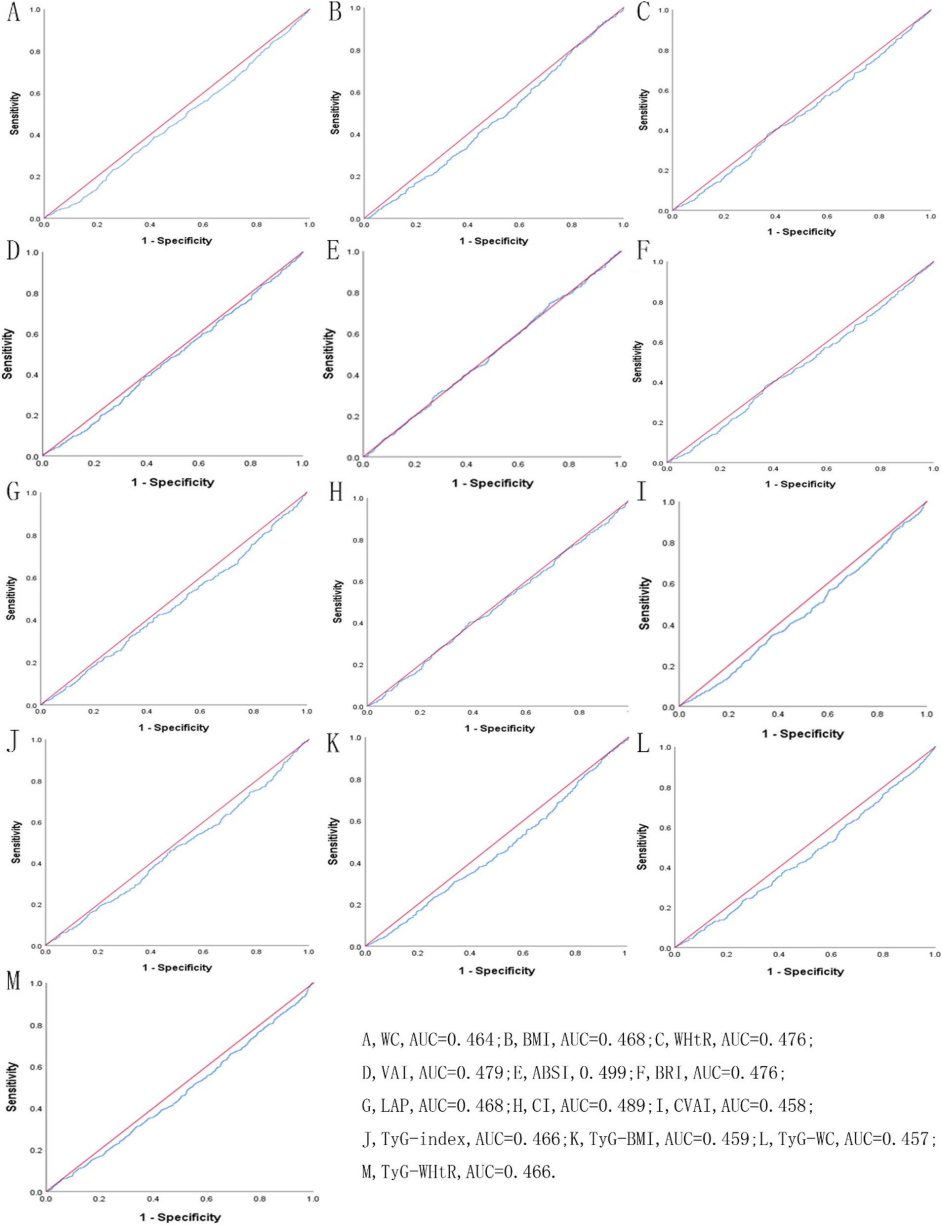
^The ROC curves of each indicator in the prediction of depressive symptoms risk in females at 2011→2015^

Table 5 showed the cut-off between area under curve, sensitivity, and specificity for obesity- and lipid-related indices to detect subsequent onset of depressive symptoms by sex. The ROC curves of each index for predicting depressive symptoms risk in males and females are shown in Fig. 1 and Fig. 2 during the short-term, Fig. 3 and Fig. 4 during the long-term, respectively. In males, WHtR (AUC = 0.462, SE = 0.017, 95% CI = 0.429–0.495, and optimal cut-off = 0.432) and BRI (AUC = 0.462, SE = 0.017, 95% CI = 0.429–0.495, and optimal cut-off = 2.176) had the largest predictive values among 13 indicators during the short-term (*P* < 0.05). In females, BMI (AUC = 0.468, SE = 0.015, 95% CI = 0.439–0.496, and optimal cut-off = 19.378) and LAP (AUC = 0.468, SE = 0.015, 95% CI = 0.439–0.497, and optimal cut-off = 2.163) had the largest predictive values among 13 indicators during the long-term (*P* < 0.05). However, there was no significant predictive ability of ABSI for depressive symptoms in both males and females during both follow-up periods (*P* > 0.05).

**Table 5:** Cut-off between area under curve, sensitivity, and specificity for obesity- and lipid-related indices to detect subsequent onset of depressive symptoms by sex

**Follow-up periods**		**WC**	**BMI**	**WHtR**	**VAI**	**ABSI**	**BRI**	**LAP**	**CI**	**CVAI**	**TyG index**	**TyG-BMI**	**TyG-WC**	**TyG -WHtR**
**2011 → 2013**	**Male**													
***N* = 3790**	Area under curve	0.452	0.467	0.462	0.461	0.468	0.462	0.450	0.460	0.449	0.470	0.461	0.451	0.456
	Std. Error	0.017	0.017	0.017	0.017	0.017	0.017	0.017	0.017	0.017	0.017	0.017	0.017	0.017
	95%CI	0.420,0.485	0.433,0.500	0.429,0.495	0.428,0.494	0.435,0.501	0.429,0.495	0.417,0.483	0.427,0.493	0.416,0.482	0.438,0.503	0.428,0.493	0.418,0.483	0.423,0.488
	*P*-value	0.006	0.054	0.027	0.023	0.062	0.027	0.003	0.018	0.003	0.085	0.022	0.004	0.010
	Optimal cutoffs	105.800	17.890	0.432	1.067	9.512	2.176	2.330	1.463	11.415	7.830	140.453	572.583	3.562
	J-Youden	0.002	0.005	0.004	0.010	0.015	0.004	0.010	0.009	0.003	0.035	0.005	0.009	0.012
	Sensitivity (%)	2.9%	97.1%	96.8%	92.9%	2.6%	96.8%	99.7%	1.8%	99.4%	95.6%	98.8%	97.1%	95.6%
	Specificity (%)	97.3%	3.4%	3.6%	8.1%	98.9%	3.6%	1.3%	99.1%	0.9%	7.9%	1.7%	3.8%	5.6%
	( +) Likelihood ratio	1.074	1.005	1.004	1.011	2.364	1.004	1.010	2.000	1.003	1.038	1.005	1.009	1.013
	(-) Likelihood ratio	0.998	0.853	0.889	0.877	0.985	0.889	0.231	0.991	0.667	0.557	0.706	0.763	0.786
	**Female**													
	Area under curve	0.478	0.472	0.491	0.490	0.514	0.491	0.486	0.502	0.483	0.499	0.480	0.483	0.491
	Std. Error	0.016	0.016	0.016	0.016	0.016	0.016	0.016	0.016	0.016	0.016	0.016	0.016	0.016
	95%CI	0.447,0.509	0.440,0.503	0.460,0.522	0.460,0.521	0.483,0.545	0.460,0.522	0.455,0.517	0.471,0.533	0.453,0.514	0.468,0.530	0.448,0.511	0.452,0.514	0.460,0.522
	*P*-value	0.160	0.072	0.560	0.545	0.387	0.559	0.382	0.898	0.288	0.946	0.196	0.282	0.575
	Optimal cutoffs	111.300	28.450	0.498	1.732	8.222	3.317	51.734	1.249	47.638	9.828	255.828	778.741	5.243
	J-Youden	0.003	0.026	0.014	0.024	0.045	0.014	0.007	0.035	0.017	0.019	0.011	0.013	0.012
	Sensitivity (%)	0.9%	15.0%	84.2%	93.8%	60.3%	84.2%	31.5%	74.3%	93.3%	7.6%	15.4%	41.1%	33.3%
	Specificity (%)	99.4%	87.6%	17.2%	8.6%	44.2%	17.2%	69.2%	29.2%	8.4%	94.3%	85.7%	60.2%	67.9%
	( +) Likelihood ratio	1.500	1.210	1.017	1.026	1.081	1.017	1.023	1.049	1.019	1.333	1.077	1.033	1.037
	(-) Likelihood ratio	0.997	0.970	0.919	0.721	0.898	0.919	0.990	0.880	0.798	0.980	0.987	0.978	0.982
**2011 → 2015**	**Male**													
***N* = 3660**	Area under curve	0.478	0.478	0.489	0.504	0.500	0.489	0.490	0.492	0.484	0.499	0.480	0.484	0.491
	Std. Error	0.016	0.016	0.016	0.016	0.017	0.016	0.016	0.016	0.016	0.016	0.016	0.016	0.016
	95%CI	0.446,0.509	0.446,0.510	0.457,0.520	0.472,0.536	0.467,0.532	0.457,0.520	0.458,0.522	0.460,0.524	0.453,0.516	0.467,0.531	0.448,0.512	0.453,0.516	0.459,0.523
	*P*-value	0.163	0.164	0.471	0.822	0.984	0.471	0.524	0.614	0.318	0.947	0.200	0.326	0.562
	Optimal cutoffs	99.050	24.648	0.580	5.873	8.471	4.980	45.22	1.339	161.812	8.984	229.898	922.397	5.366
	J-Youden	0.026	0.007	0.015	0.051	0.058	0.015	0.042	0.055	0.032	0.047	0.028	0.029	0.031
	Sensitivity (%)	11.40%	32.00%	17.10%	22.00%	31.00%	17.10%	24.40%	23.00%	13.30%	29.60%	24.40%	10.90%	14.50%
	Specificity (%)	91.20%	68.70%	84.40%	83.10%	74.80%	84.40%	79.80%	82.50%	89.90%	75.10%	78.40%	92.00%	88.60%
	( +) Likelihood ratio	1.295	1.022	1.096	1.301	1.230	1.096	1.208	1.314	1.317	1.189	1.130	1.363	1.272
	(-) Likelihood ratio	0.971	0.990	0.982	0.939	0.922	0.982	0.947	0.933	0.964	0.937	0.964	0.968	0.965
	**Female**													
	Area under curve	0.464	0.468	0.476	0.479	0.499	0.476	0.468	0.489	0.458	0.466	0.459	0.457	0.466
	Std. Error	0.015	0.015	0.015	0.015	0.015	0.015	0.015	0.015	0.015	0.015	0.015	0.015	0.015
	95%CI	0.435,0.493	0.439,0.496	0.447,0.505	0.450,0.507	0.470,0.528	0.447,0.505	0.439,0.497	0.460,0.518	0.429,0.487	0.437,0.495	0.430,0.487	0.428,0.486	0.437,0.495
	*P*-value	0.015	0.028	0.107	0.148	0.952	0.107	0.029	0.467	0.004	0.023	0.005	0.004	0.021
	Optimal cutoffs	109.800	19.378	0.583	40.516	7.985	5.039	2.163	1.319	2.422	7.522	150.143	495.547	3.269
	J-Youden	0.003	0.009	0.011	0.006	0.024	0.011	0.003	0.016	0.002	0.005	0.002	0.004	0.006
	Sensitivity (%)	1.40%	93.10%	38.10%	1.00%	74.60%	38.10%	100.00%	40.20%	100.00%	98.80%	96.60%	99.60%	99.40%
	Specificity (%)	98.90%	7.80%	63.00%	99.60%	27.80%	63.00%	0.30%	61.40%	0.00%	1.70%	3.60%	0.40%	0.60%
	( +) Likelihood ratio	1.273	1.010	1.030	2.500	1.033	1.030	1.003	1.041	1.002	1.005	1.002	1.004	1.006
	(-) Likelihood ratio	0.997	0.885	0.983	0.994	0.914	0.983	0.000	0.974	0.000	0.706	0.944	0.000	0.000

*WC* waist circumference, *BMI* body mass index, *WHtR* waist to height ratio, *VAI* visceral adiposity index, *ABSI* A body shape index, *BRI* body roundness index, *LAP* lipid accumulation product, *CVAI* Chinese visceral adiposity index, *CI* conicity index, *TyG* triglyceride glucose index, *TyG-BMI* TyG related to BMI, *TyG-WC* TyG related to WC, *TyG-WHtR* TyG related to WHtR

## Discussion

In our nationwide cohort study, we used ROC analysis to determine the predictive power of obesity- and lipid-related indicators for depressive symptoms. Our findings revealed that the AUC values of most indicators are below 0.5, indicating that the discriminative power of these indices is weak and not significantly better than random chance [64]. Although many previous studies [31, 65, 66] have reported a relationship between obesity and depressive symptoms, almost no research has investigated the predictive ability of indicators for depressive symptoms. Our study for the first time investigated the predictive ability of 13 indicators for depressive symptoms based on cohort studies, and found that all indicators had limited predictive ability for depressive symptoms.

We also found the incidence of depressive symptoms in females was 25.44% in short-term follow-up and 34.30% in long-term follow-up, consistently higher than in males during both follow-up periods. This is consistent with previous published studies [17, 67, 68]. Due to hormonal fluctuations (such as excessive sensitivity to hormonal fluctuations and menopausal hormonal changes), endocrine disorders can occur, making women prone to emotional fluctuations, depression, and reluctance to interact with others [69]. In, addition, psychosocial events, victimization, gender specific socialization, internalized coping strategies, and disadvantaged social status, females may be more prone to depression than males [70, 71]. From the perspective of social differences, women may experience more stressful life events throughout their lives, and they are more sensitive to these events [70]. When faced with trouble, there is a significant difference in coping styles between women and men. Women are more inclined to focus on the emotions and repetitive thinking caused by problems, and this reflective coping style may lead to a higher incidence of depression.

Moreover, the significantly negative associations were found between depressive symptoms and most obesity- and lipid-related indicators, but differed by sex (male, female) and length of follow-up (2 years, 4 years). In males, the significant association between depressive symptoms and WC, LAP, CVAI, TyG-WC was observed in the short-term, but not observed in the long-term. No association between BMI and depressive symptom was observed in males during both follow-up periods. This could be because BMI is only a surrogate measure of body fatness and does not distinguish body composition (muscle and fat accumulation), especially for males who often have more muscle mass and less fat mass than females [18]. Therefore, if only BMI is considered, males may be more susceptible to these limitations. In females, the significant association between depressive symptoms and WC, TyG-WC was observed in the short-term, and WC, BMI, WHtR, BRI, LAP, CVAI, TyG index, TyG-BMI, TyG-WC, and TyG-WHtR was observed in the long-term. Compared to short-term follow-up, our study found that more indicators showed a significant negative correlation with depressive symptoms in females during long-term follow-up, which can be explained by cumulative effects.

However, we did not find the significant association between ABSI and depressive symptoms. Unlike our results, Lotfi K, Hassanzadeh Keshteli A, Saneei P, et al. found that ABSI was positively related to odds of depressive symptoms measured by the Hospital Anxiety and Depression Scale among Iranian females but not in males [72]. There are several points that can explain the differences between our survey results and the results of the aforementioned survey report. Firstly, previous research was conducted among Iranian adults, while our survey was conducted among the middle-aged and elderly population in China, with differences in demographic characteristics such as race and age. Secondly, Lotfi, K et al*.* used the Hospital Anxiety and Depression Scale. However, we used the Chinese version of the CES-D scale in our study, and there were differences in the diagnostic criteria for depression between the two measurement tools. Thirdly, previous research was a cross-sectional study, while ours is a cohort study with a larger sample size and analyzed the predictive ability of ABSI, therefore the current study has greater ability to detect these relationships. According to ROC analysis, the results for the ABSI AUC did not reach statistical significance in males and females during both follow-up periods (*P* > 0.05), respectively. Hence, ABSI was not a valuable predictive indicator of depressive symptoms for both males and females.

According to our results, we supported the “fat and jolly” hypothesis in middle-age and elderly Chinese, in consistent with many previous studies [27, 29, 73–75]. Crisp AH, et al*.* first reported the "jolly fat" hypothesis in a middle-aged sample of the general population, which suggests a significant positive correlation between severe obesity in men and low levels of depression [75]. In addition, Yim G, Ahn Y, Cho J, et al. also found the association of obesity and depressive symptoms in 2210 Korean middle-aged women, supporting the “jolly fat” hypothesis, which suggests that women with general obesity were less likely to have depressive symptoms [74]. However, some cross-sectional studies suggest a positive correlation between obesity and depression [67, 68]. Part of the reasons for the differences may be due to cultural differences, as people in different regions have different attitudes towards obesity. Weight bias is very common in American society. According to a survey, the prevalence of weight bias in the United States has increased by 66% in the past decade [76]. Weight stigmatization may be one of the risk factors for depression in obese individuals, and weight-based ridicule has been found to be a mediating factor in the relationship between obesity and depression [77]. A review summarizes evidence that internalization of weight bias is associated with negative mental health outcomes such as depression, anxiety, inferiority complex, and quality of life [78]. But in Chinese cultural tradition, the connection between happiness and obesity is described by a famous idiom " happy mind and fat body " [79]. Compared to Western culture, Chinese people believed that obesity is not a symbol of unhealthy behavior, as only wealthier people can afford more food and gain weight. In addition, middle-aged weight gain is considered a good omen of good luck, so people are willing to gain weight in their later years [28].

### Strengths and limitations of the study

The main strength of our study are as follows: Firstly, we analyzed data based on a nationwide population-based longitudinal study. This study enrolled 3790 and 3660 middle-aged and elderly Chinese individuals in both short-term and long-term follow-up, the large sample size enhanced the generalizability and effectiveness of the research results. Secondly, it evaluated the impact of obesity- and lipid- related indicators on the depressive symptoms throughout two different interval periods. It helps us understand the short-term and long-term effects of 13 indicators on the incidence of depressive symptoms.

The study has several limitations should be noted. Firstly, depression symptoms were measured using the CES-D self-report scale, which has been shown to have acceptable psychological measurement characteristics and is suitable for a wider range of elderly participants. However, due to people tend to underreported their mental disease in the research, there may be reporting bias in the results. Secondly, with the deepening of aging, the incidence rate of depressive symptoms among middle-aged and elderly people is rising, which is a serious problem facing China. Therefore, this study included middle-aged and elderly people aged 45 and above in China. It is worth noting that the results of our study in the context of other age groups should be interpreted with caution. Lastly, our results indicate that the AUC values of most indicators are below 0.5, indicating low diagnostic accuracy and inability to effectively predict depression in clinical practice. In future research, we need to try to combine two or more indicators to see if it can improve diagnostic accuracy.

## Conclusion

Among the obesity- and lipid-related indices, ABSI did not correlate with depressive symptoms and failed to serve as a valuable predictor for both males and females across all intervals. Our research findings indicate that most obesity- and lipid-related indicators have statistical significance in predicting depressive symptoms, but the accuracy of these indicators in prediction is relatively low and may not be practical predictive factors. The results of this study may be of great significance for the early identification and prevention of depressive symptoms in middle-aged and elderly Chinese. Given the urgency of early screening for high-risk individuals for depressive symptoms, future research can explore the use of multiple indicators in combination to test whether they can improve the predictive ability of depressive symptoms, and thus have practical applications in clinical practice.

## Data Availability

Data can be accessed via http://opendata.pku.edu.cn/dataverse/CHARLS.

## Abbreviations

CHARLS: China Health and Retirement Longitudinal Study
WC: Waist circumference
BMI: Body mass index
WHtR: Waist-height ratio
VAI: Visceral adiposity index
ABSI: A body shape index
BRI: Body roundness index
LAP: Lipid accumulation product
CI: Conicity index
CVAI: Chinese visceral adiposity index
TyG index: Triglyceride glucose index
TyG-BMI: Triglyceride-glucose related to BMI
TyG-WC: Triglyceride-glucose related to WC
TyG-WHtR: Triglyceride-glucose related to WHtR
CES-D: The Chinese version of the Center for Epidemiologic Studies Depression scale
ROC: Receiver operating characteristic curve
AUC: Area under curve
SPSS: Statistical Product Service Solutions
ORs: Odds ratios
Cis: Confidence intervals
SE: Standard error
